# Wild Foundress Queen Bumble Bees Make Numerous, Short Foraging Trips and Exhibit Frequent Nest Failure: Insights From Trap‐Nesting and RFID Tracking

**DOI:** 10.1002/ece3.71016

**Published:** 2025-02-21

**Authors:** Erica Sarro Gustilo, William H. Grover, S. Hollis Woodard

**Affiliations:** ^1^ University of California Riverside California USA

**Keywords:** Bombus, bumble bee, foraging, foundress, queen, RFID, survival

## Abstract

The overwhelming majority of research on wild bumble bees has focused on the social colony stage. Nest‐founding queens in the early season are difficult to study because incipient nests are challenging to find in the wild and the foundress queen flight period is very short relative to the entire nesting period. As a result, natural history information on foundress queens is exceedingly rare. New methodological approaches are needed to adequately study this elusive life stage. We trap‐nested wild queen bumble bees in artificial nest boxes in Gothic, Colorado and used a custom‐built radio frequency identification (RFID) system to continuously record queen foraging activity (inferred from entering and exiting the nest) for the majority of their spring flight periods. Foundress queens made frequent, short foraging trips, which tended to increase in duration over the course of the flight period. All queens who produced adult workers ceased foraging within approximately 1 week after workers emerged in the nest. We observed frequent nest failure among foundress queens: Fewer than one quarter of queens who laid eggs in nest boxes went on to produce reproductive gynes at the end of the season. We also report nest characteristics and curious phenomena we observed, including conspecific nest invasion and queens remaining outside the nest overnight. We present this trap‐nesting and subsequent RFID tracking method as a valuable, albeit resource‐intensive, path forward for uncovering new information about the elusive, incipient life stage of wild bumble bees.

## Graphical abstract

Using a trap‐nesting and RFID tracking methodology, we report wild bumble bee queen foraging into and out of the nest as well as success rates of foundress queens. Queens made frequent, short foraging trips and ceased foraging shortly after workers emerged in the nest. Fewer than one quarter of queens observed in our study succeeded in producing reproductive queens (gynes) at the end of the season.

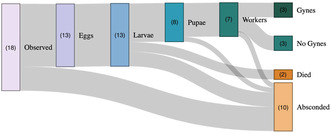

## 1 Introduction

The bumble bees (genus *Bombus*, family Apidae) are a group of generalist pollinators found worldwide, particularly in more temperate and montane areas (Goulson 2003). Most species in the genus, except for parasitic species and some social species found in the tropics, have an annual eusocial lifestyle in which queens found nests independently each spring after overwintering in a state of diapause (Alford 1969). After queens initiate egg laying, their first offspring (typically workers, the largely non‐reproductive female caste) begin to emerge several weeks later in the nest (Goulson 2010). Colonies then grow to contain anywhere from fewer than 10 to up to several hundred workers, who carry out the majority of the work (e.g., foraging) for the nest (Goulson 2010). If nests successfully reach maturity, they will produce reproductives (gynes and males) that leave the nest before it ultimately dies at the end of the season (Alford 1975).

The overwhelming majority of wild bumble bee research has focused on the worker caste during the social colony stage wherein nests contain multiple cohorts of workers. The primary reason for this bias is that workers are more readily encountered (often, foraging on flowers) and are observed over a longer duration in the active flight season than queens or males, whose flight periods are much more ephemeral. Other stages in the bumble bee life cycle are more difficult to study. In particular, the queen overwintering (Williams et al. 2019; Pugesek et al. 2023) and nest‐founding (Kells and Goulson 2003; Pugesek and Crone 2022) stages have proven challenging to study in the wild due to the difficulty of locating bees during these periods. For example, wild bumble bee nests, which are often underground, are generally difficult to find (Liczner and Colla 2019; Liczner et al. 2021) and are even more so when they consist of only a single adult queen. This leaves us to study these life stages largely in a laboratory setting, which limits our understanding of the ecological and other processes that shape them (but see, e.g., Sladen 1912; Richards 1973; Richards 1978; Williams et al. 2019; Miller et al. 2023).

Ultimately, new methodological advances are needed to study wild bumble bees during their more difficult‐to‐study life stages (Mola and Williams 2019; Williams et al. 2019; Makinson et al. 2019). We developed a methodology to trap‐nest and monitor nest‐founding (“foundress”) queen bumble bees with a custom‐built radio frequency identification (RFID) system. We monitored wild nest establishment in artificial nest boxes deployed around the Rocky Mountain Biological Laboratory in Gothic, Colorado, and continuously recorded queen comings and goings from their nests for the majority of their spring flight periods with RFID tracking. Our primary goals were to gain insight into the survival, nesting success, and foraging activity of wild foundress queens. Gaining knowledge about foraging activity during nest‐founding can shed light on how queens may juggle the various demands on their time, energy, and other resources at this life stage (Gustilo et al. 2023). Here, we provide general insights into the biology and natural history of nest‐founding bumble bee queens that were generated using our methodology and present it as an auspicious approach to studying this elusive, yet foundational life stage in bumble bees.

## 2 Materials and Methods

### 2.1 Study Sites

We placed 100 wooden nesting boxes (~20 × 20 × 20 cm; ½″ maple sanded SoyStrong plywood with one 1.5 cm entrance hole on the side of each box) at subalpine sites at the Rocky Mountain Biological Laboratory (Gothic, Colorado) in early spring of 2021 (around May 15) and left them out through fall of 2022 (around September 30) to encourage *Bombus* queen colonization in the springs of 2021 and 2022.

We intentionally varied nest box locations and box characteristics in a factorial design, in an effort to identify factors that may influence queen colonization and improve colonization rates in future work. We placed boxes in one of five landscape types (willow, aspen, conifer, open meadow, and within 1 m of a wood cabin), either entirely within the landscape or within 2 m of the edge of the landscape. Bumble bee species found in this region are known to nest underground, in tree cavities, and in other cavities on the ground surface (Liczner and Colla 2019); thus, we placed boxes either directly on the ground or strapped to trees or other structures at a height of approximately 1.5 m. We avoided burying boxes underground to enable access to the nest interior for queen tagging and brood monitoring (details below). Bumble bees typically nest in cavities that have existing insulation materials (Heinrich 2004); thus, we filled boxes with small animal bedding, including aspen wood shavings, sterilized sphagnum moss, and paper bedding according to Mjelde (2020), with the exception that we omitted all cotton substrate, which can get caught on radio frequency identification tags. There is some evidence that bumble bees may be attracted to rodent nest material (Varner et al. 2023); thus, we lined the interior of some boxes with materials from abandoned rodent nests found within Gothic, in addition to the above insulation materials. We also added baked clay balls (1 cm diameter) to half of all nest boxes. Laboratory‐reared queens prefer to lay eggs on these clay balls (Gustilo and Woodard 2020); thus, we included them in the event that wild queens might also find them attractive. We covered some of the nest boxes with clear plastic sheeting to repel rain and pre‐attached the RFID tunnels (described below) to some boxes. Sample sizes for each nest box characteristic can be found in Table A1. Low nest box selection rates precluded statistical analyses on nest box characteristics.

### 2.2 Study Subjects

**FIGURE 1:**
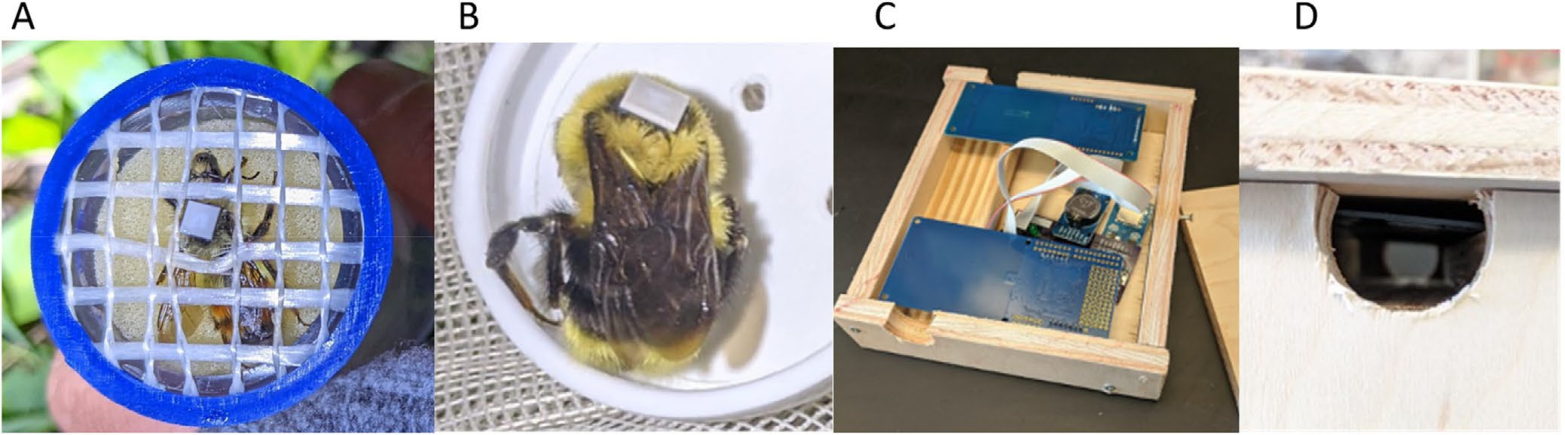
Photos of RFID system. (A) Queen tagged in queen marking tube (B) Queen with RFID tag glued to thorax. (C) Paired RFID antenna cards (blue rectangles) connected to a central custom PCB with a real‐time clock and microSD card slot. All bees had to walk through a channel beneath the RFID antenna cards (D) in order to enter or exit their nest box.

From early May through the end of June in both 2021 and 2022, we inspected nest boxes weekly, at night, to check for evidence of queen colonization. To do so, we inserted an endoscopic camera (Anhendeler B315) into the nest entrance while watching the video feed as well as listening for buzzing sounds coming from the nest. We considered a nest successfully colonized when a bumble bee queen and brood wax were present in the nest. We tagged queens within 24 h of observing nest colonization. We tried to tag queens as early as possible in their nesting period, though we occasionally did not discover colonization until later in the nesting period (possibly because the queen diverted the insulation material at the nest entrance and our camera was routed away from the nest). As a result, nests varied in their developmental stage at the time of queen tagging (ranging from first clutch of eggs to multiple adult workers), though most queens had only young larvae and eggs at the time of tagging (Table A2). To tag each queen, we temporarily removed queens from nest boxes and placed them into queen marking tubes (Figure 1). Queens were not anesthetized for this process to avoid unintentionally harming them. While queens were immobilized in the marking tube, we identified them to species and attached an RFID tag (3.2 × 3.2 × 0.4 mm; 0.099 g; Murata Electronics XMS33HCNK‐171) to their thorax with cyanoacrylate glue (Figure 1). We then affixed a custom‐built bidirectional RFID reader (described below) to the nest entrance to passively collect timestamp data as the queen entered and exited the nest (Figure 1).

For several weeks after the queens were tagged, we monitored colonized nest boxes twice weekly, during the day, to replace RFID reader batteries. We disturbed nests as little as possible to avoid inadvertently encouraging nest abandonment. In 2022, we also conducted daily, hour‐long, in‐person observations from the day that individual queens were tagged until 2 days after they ceased foraging. During these observations, we recorded the time of all instances that queens or workers exited or entered the nest and the caste of the individual. This was done to validate that our RFID data were consistent with queen foraging data and to identify the date that workers began foraging in each nest. In 2021, we did not monitor for the presence of workers in a standardized fashion; thus, the date reported as nests first having workers refers to the first date that workers were observed in the nest; workers may have emerged in these nests earlier than observed dates (up to an estimated 7 days).

We removed RFID readers and ceased monitoring > 3 weeks and > 48 h after queens ceased foraging at a given nest (based on both in‐person and RFID records) in 2021 and 2022, respectively. In 2022, we also opened nest boxes under red light at night > 48 h after the last observation of queen foraging to verify the queen was still present, count the number of adult workers, and note the stage of any brood in the nest (eggs, larvae, and pupae).

**FIGURE 2:**
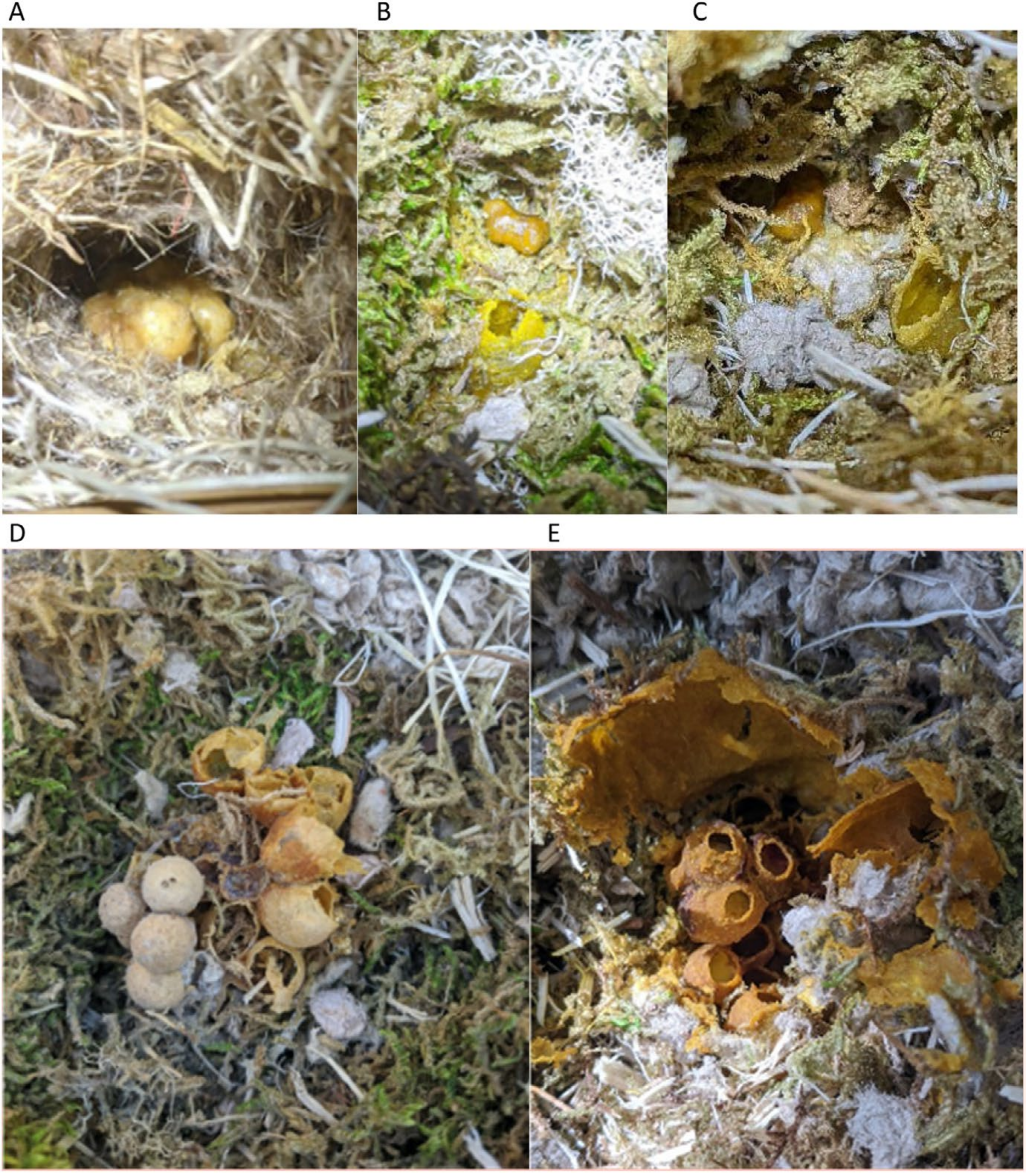
Photos of colonized nest boxes. (A–C): Nests at the time of queen tagging; (A) *B. appositus* mature larvae and honeypot; (B) *B. rufocinctus* young larvae and honeypot; (C) *B. rufocinctus* young larvae and honeypot. (D–E): Nests at the end of the season with no living bees remaining; (D) *B. centralis* ; and (E) *B. rufocinctus* . All nests were enclosed in nesting materials, which were pulled back to expose the brood wax in these photos. The *B. rufocinctus* nest in (E) was also covered in a waxen canopy, which was also pulled back prior to photographing.

We returned to sites in late September of each year, after the nesting season had ended, to dissect colonized nest boxes and quantify gyne production as a metric of reproductive output in nests (Figure 2). Queens of most species are approximately 1.5 times the size of workers of the same species (del Castillo and Fairbairn 2012), and this body size difference is reflected in pupal case sizes (Elliott 2009). Thus, we measured the number and size of intact, empty pupal cases in our study nests to quantify colony reproductive output (i.e., estimated total number of new queens produced based on queen pupal casings) over the course of the season. Some worker and/or queen pupal casings may have been repurposed into honey pots within nests; thus, our counts may be underestimates.

### 2.3 RFID Technology

RFID readers consisted of a custom‐made printed circuit board (PCB) connected to two antenna cards (Adafruit PN532). An onboard lithium‐ion button‐cell battery powered a 24‐h clock, and a microSD card slot transferred RFID read data to external storage. Each paired reader was powered by a portable 6 V battery. The antenna cards were positioned over a tunnel through which all bees had to walk to enter or exit the nest (Figure 1). In this way, the RFID tag on a bee's thorax was designed to come in close proximity with the antenna cards upon every entrance and exit to and from the nest, at which point a timestamp record with the unique RFID tag identifier was automatically printed onto the external microSD card.

### 2.4 Data Filtering and Analyses

All data filtering and analyses were conducted in R version 4.0.3 (Team RC 2020). It must be cautioned that our RFID system was not foolproof. It is possible that queens entered or exited the nest without the RFID system picking up on their movement; for example, if bees entered the nest upside down with the RFID tag pointing away from the reader, or for particularly small queens whose thorax was a distance below the reader as they walked through, the reader may not detect passage. This may have resulted in an overestimation of the duration of some foraging bouts and an underestimation of the number of foraging bouts overall. To quantify and account for this potential source of error, we compared 66.35 h of RFID data to concurrent in‐person observations.

We filtered the raw RFID reads based on several assumptions. First, we defined paired reads as successive reads on opposite boards with < 10 s between reads. Next, we labeled the direction of motion for each paired read as either an “entrance” or “exit” from the nest, based on the order of reads (i.e., reads on the inner board followed by the outer board were labeled as an “exit,” and vice versa labeled as an “entrance”). We then identified lone, unpaired reads that occurred > 10 s before or after any other reads. For each unpaired read, if it was immediately preceded by and immediately followed by an entrance to the nest, it was relabeled as an exit. Likewise, if it was immediately preceded by and immediately followed by an exit from the nest, it was relabeled as an entrance. Then, we calculated the length of time between each entrance or exit and the subsequent read, to identify the duration of each out‐of‐nest (presumably, foraging) trip. We removed all remaining unpaired reads on the inner reader board (closer to the nest) that were preceded by an entrance or another unpaired inner board read (*n* = 32 reads across all queens); these were likely the result of the queen standing in the nest entrance without actually leaving. We also removed trips outside the nest lasting less than 30 s (*n* = 49 records across all queens), which were likely the result of defecation trips (Dosselli et al. 2016) or queens standing in the nest entrance without actually leaving the nest. Finally, we removed any trips interrupted by a battery change on the RFID system because any loss of power during the study could have resulted in missed reads and invalid trip duration.

To summarize foraging behavior within and among individuals, we calculated summary statistics (mean and standard error [s.e.m.]) of foraging bout duration and the number of foraging bouts per day for each nest. We compared foraging activity for queens in the early foundress stage to those in the late foundress stage using mixed models. We define the early stage as the first 5 days of foraging for queens who had larvae in the nest at the time of tagging and the late stage as the last 5 days of foraging for queens who successfully produced workers. To analyze foraging duration, we used a linear mixed model with log‐transformed foraging duration as the predictor variable, stage (early or late) as a fixed effect, and queen identity as a random effect. To analyze foraging frequency, we used a generalized linear mixed model with a negative binomial distribution with foraging frequency as the predictor variable, stage (early or late) as a fixed effect, and queen identity as a random effect. We included only queens for which we recorded both early and late stage foraging in these stage‐based analyses (*n* = 6 queens). All models were checked for overdispersion. We created all data visualizations with the ggplot2 package (Wickham 2016) in R.

## 3 Results

### 3.1 Selection and Colonization of Nest Boxes

Here, we define *colonized* boxes as those in which a queen laid eggs and formed at least one honeypot, which is a behavior typically observed in nest founding queens (Alford 1975; Heinrich 2004). A total of 13 queens of five species commonly observed in the study area colonized nest boxes across both years (Table A2). In all colonized boxes, queens constructed honeypots adjacent to the brood clump and between the brood and the nest entrance, as described for various species in Alford (1975) and Heinrich (2004) (Figure 2). No queens laid eggs on provided clay balls.

**FIGURE 3:**
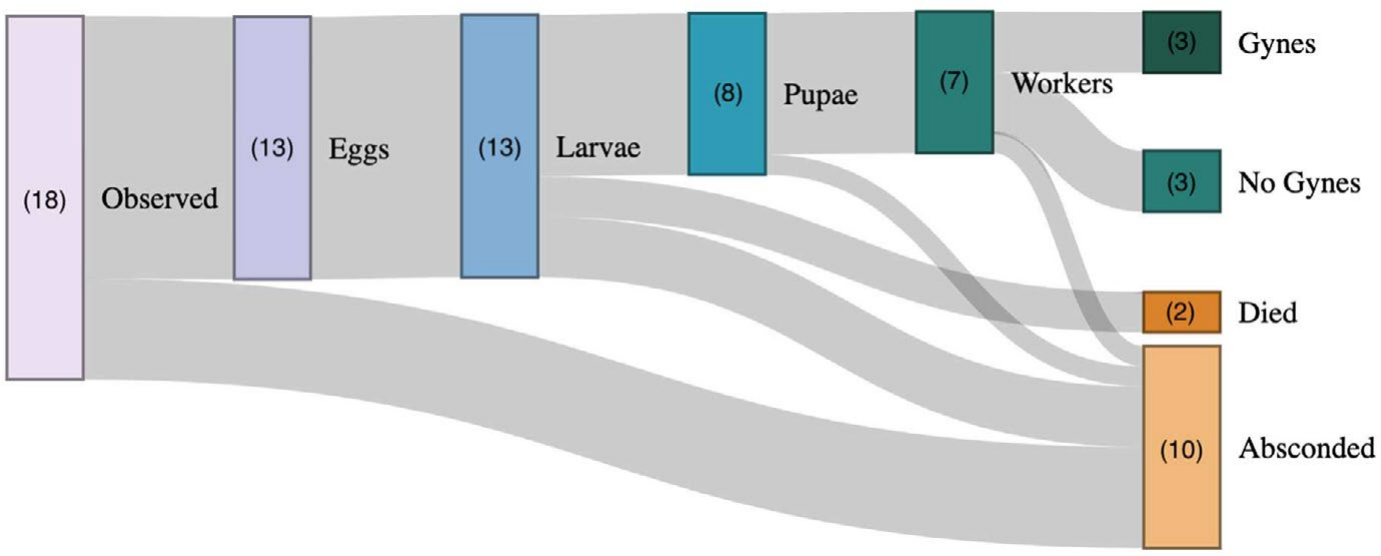
Fate of all queens observed in the study. Numbers in parentheses represent the number of queens we observed in each stage. Links between nodes represent the progression of queens. The three queens who successfully produced gynes at the end of the season (“Gynes”) comprised approximately one sixth (16%) of all queens observed in nest boxes (“Observed”), just under one quarter (23%) of queens who laid eggs (“Eggs”), and less than half (42%) of queens who produced adult workers (“Workers”).

We observed an additional five queens that we refer to as having *selected* nest boxes, meaning that they entered nest boxes (and were observed in them at night) in 2022 but absconded (i.e., departed for an unknown reason and did not return) from nest boxes prior to laying eggs or forming a honeypot (*n* = 1 *B. bifarius* ; *n* = 4 unknown species; Table A2; Figure 3). No queens in 2022 selected or colonized boxes that were previously colonized in 2021.

### 3.2 Queen Survival and Nest Development

Given the small sample size of RFID‐tagged queens in our study and low within‐species replication (nine queens of five species), we report subsequent results descriptively and with the goal of providing some general insights into the patterns of early‐nesting queen survival, nest development, and foraging activity.

Of the 13 queens who colonized boxes, approximately half (*n* = 7) produced adult workers (Figure 3). The remaining six queens either absconded from the nest (*n* = 4) or died in the nest box after their eggs developed into larvae or pupae but before they matured into adult workers (*n* = 2) (Figure 3). Absconding from the nest may represent a choice on the part of the queen or queen death due to predation or some other factor. Of the two queens who died in the nest boxes, one queen's nest box was overrun by ants and the second was tagged on a particularly cold night; although we have never observed mortality from this RFID tagging process in previous projects (*n* = > 50 wild‐caught and laboratory‐reared queens; Gustilo unpublished data), it is possible that the stress from tagging combined with the cold weather resulted in mortality.

**FIGURE A1:**
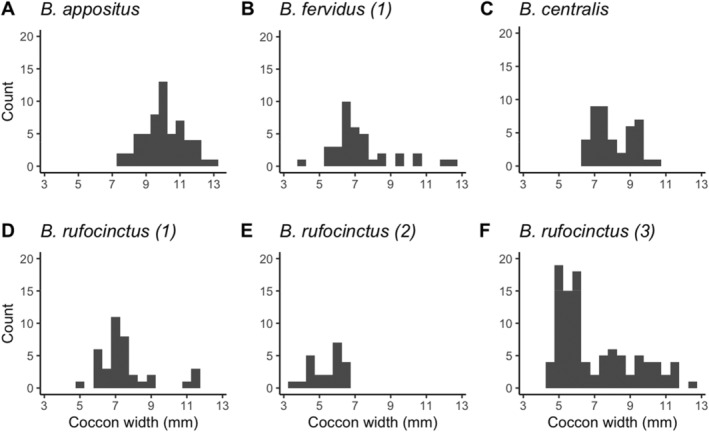
Histograms of widths of pupal cocoons at the end of the season. Bimodal distributions and a greater than twofold size difference among cocoons in B, D, and F suggest that these nests produced gynes. Narrow size distributions in A, C, and E suggest that these nests did not produce gynes.

Of the seven queens who produced workers, three successfully produced gynes (new queens) by the end of the season, as evidenced by a greater than twofold size variation in cocoon widths with a bimodal distribution (Figure A1). We are unable to determine whether these nests produced male reproductives because male cocoons are indistinguishable from those of workers.

**FIGURE A2:**
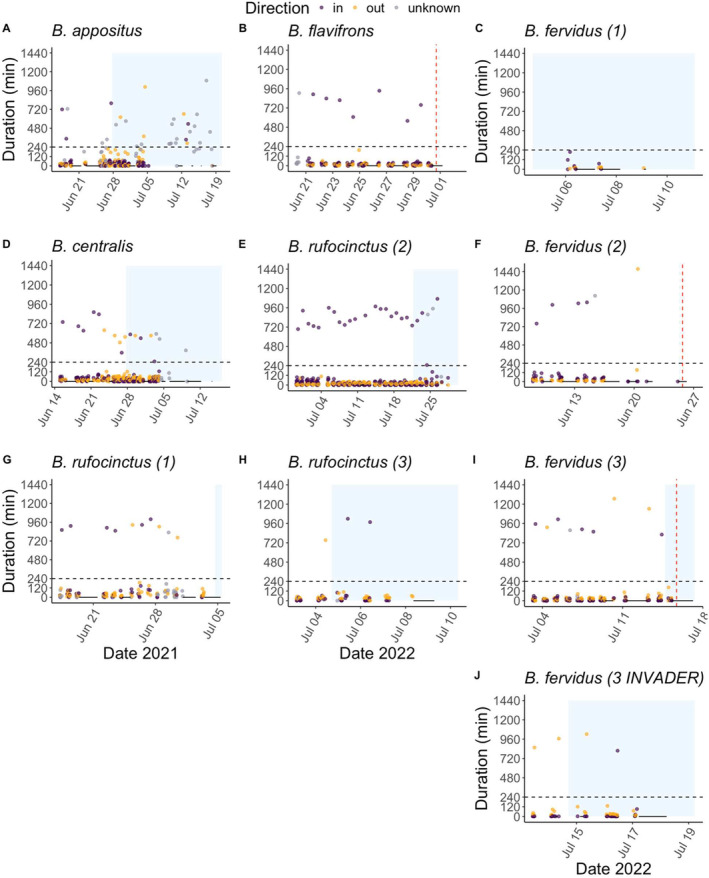
Duration of all RFID‐recorded data for all observed queens. Each point represents a recorded foraging bout. Blue rectangles represent the duration of time in which workers were observed foraging in the nest. Horizontal dashed lines represent the 4 h cutoff at which we summarized foraging trips and long duration trips separately. B and F nests never produced adult workers. 2021 nests (A, D, and G) were not monitored for workers in a standardized fashion; workers may have emerged in these nests earlier than observed dates (up to an estimated 7 days).

We observed one of the four queens who did not produce gynes abscond from the nest shortly after workers emerged, resulting in nest failure before the production of any reproductives. This particular nest box was also frequented by a second queen of the same species ( *B. fervidus* ) in the days leading up to the resident queen's absconsion. The invading queen was observed entering the nest box many times per day, and even multiple times per hour, typically remaining in the nest for 2 min or less (median 1.92 min) before leaving. We RFID‐tagged this invading queen and recorded her comings and goings from the nest for several days (Figure A2). Between in‐person and RFID‐collected observations, we recorded four consecutive days of overlap between these two queens both entering and exiting the same nest. Immediately after these 4 days and on the first day workers were observed foraging from the nest, the resident queen absconded from the nest and never returned. The invading queen continued to enter the nest for two additional days and remained in the nest overnight once before also leaving without returning.

### 3.3 Queen Foraging

**FIGURE 4:**
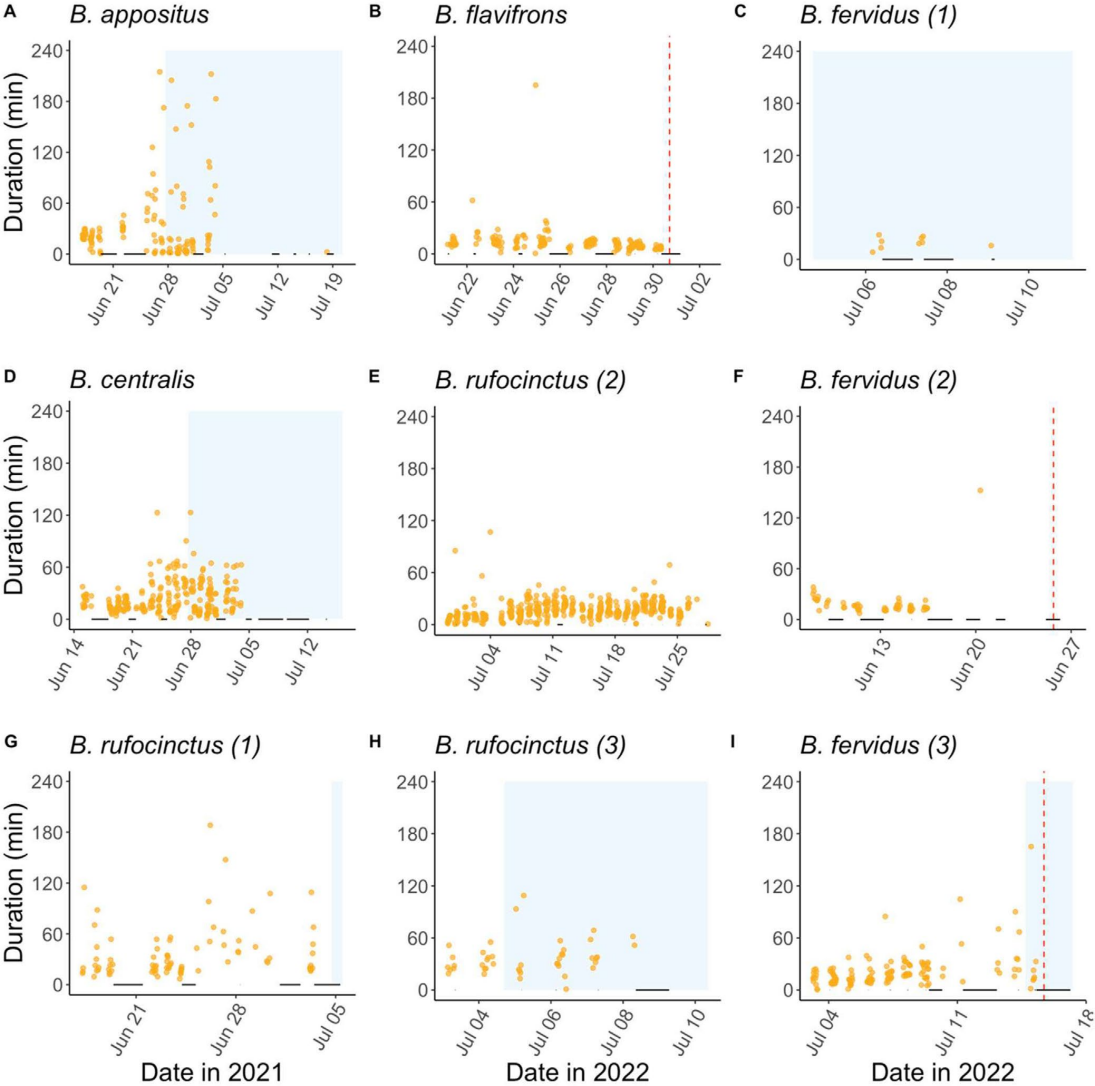
Duration of all RFID‐recorded foraging bouts of resident queens. Trips less than 30 s or greater than 4 h in duration represented less than 3% of all observations and are excluded from this figure. (A–I): Each point represents a recorded foraging bout. Black horizontal lines represent times the RFID system was not recording. Red vertical dashed lines represent queen absconsion. Blue rectangles represent the duration of time in which workers were observed foraging from the nest. B and F nests never produced adult workers. 2021 nests (A, D, and G) were not monitored for workers in a standardized fashion; workers may have emerged in these nests earlier than observed dates (up to an estimated 7 days).

RFID readers at the nest entrances continuously recorded the comings and goings of nine queens of five species from their colonized nest boxes (Figure 4; Table 1; *n* = 7 queens who successfully produced workers; *n* = 2 queens who absconded before workers emerged). Queens were each recorded for an average of 16 +/− 2.5 days, and recording ceased two or more days after queens either absconded or stopped foraging.

**TABLE 1:** Summary of resident queen foraging trips (average +/− s.e.m), as recorded by RFID readers.

Year	Species and Bee ID	Date range of recorded foraging	Number of recorded trips	Duration of trips (min)	Number of trips/day
2021	*B. appositus*	June 16–July 4	139	32.7 +/− 3.8	9.9 +/− 1.4
2021	*B. centralis*	June 14–July 4	285	24.1 +/− 1.1	15.0 +/− 1.8
2021	*B. rufocinctus* (1)	June 16–July 3	86	36.0 +/− 3.4	6.6 +/− 1.1
2022	*B. rufocinctus* (2)	June 28–July 28	476	16.6 +/− 0.5	15.9 +/− 1.3
2022	*B. rufocinctus* (3)	July 1–July 8	43	36.9 +/− 3.0	7.2 +/− 1.2
2022	*B. fervidus* (1)	July 5–July 9	9	19.6 +/− 2.1	3.0 +/− 1.0
2022	*B. fervidus* (2)	June 7–June 25	58	18.6 +/− 2.5	6.4 +/− 1.1
2022	*B. fervidus* (3)	July 1–July 15	140	21.1 +/− 1.7	11.7 +/− 2.1
2022	*B. flavifrons*	June 16–June 30	176	13.4 +/− 1.2	17.6 +/− 3.1

*Note:* Summaries exclude trips less than 30 s and greater than 4 h in duration.

**FIGURE A3:**
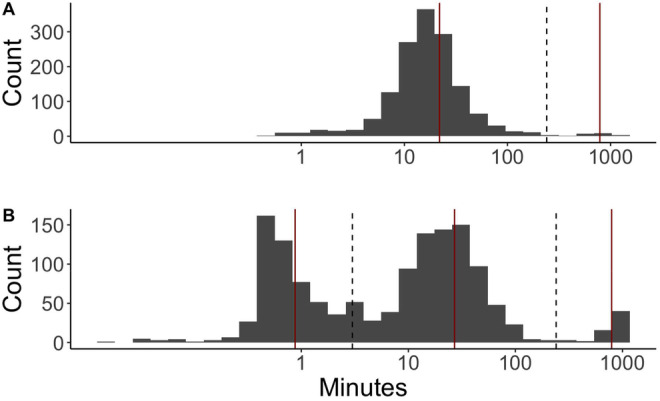
Histograms of all out‐of‐nest trip and in‐nest stay durations. *X* axes are log10 scaled to enable better visualization of spread. (A) Out‐of‐nest trips were bimodal. Vertical dashed line represents the 4 h cutoff at which we summarized foraging trips and long duration trips separately. Vertical red lines represent the mean duration of foraging trips less than 4 h (21.9 min) and trips greater than 4 h (787.7 min). (B) In‐nest stays were trimodal. Vertical dashed lines represent 3 min and 4 h cutoffs at which we summarized in‐nest stays separately. Vertical red lines represent the mean in‐nest stay duration for events less than 3 min (0.88 min), between 3 min and 4 h (27 min), and greater than 4 h (793.1 min).

We recorded 1471 trips out of the nest across all nine queens (Figure 4). The vast majority (98.5%) of trips lasted fewer than 4 h (Figure A2). We report trips less than 4 h (hereafter, “foraging trips”) and trips greater than 4 h (hereafter, “extended foraging trips”) separately, to avoid skewing summary statistics with outliers not representative of the overwhelmingly typical foraging behavior pattern. Foraging trips averaged 21.9 +/− 0.6 min with a median of 16.1 min (Table 1; Figure 4), and queens took an average of 11.8 +/− 0.7 foraging trips per day (Table 1). RFID recordings indicated that some queens occasionally remained outside of their nest boxes for more than 8 h (*n* = 21 occasions; 13.1 +/− 1.0 h), typically overnight. These extended trips happened most often in the days leading up to our observations of workers in the nest (Figure A2). Between foraging bouts, queens remained in the nest for anywhere between 1 s and nearly 18 h (Figure A3). In‐nest stay durations were trimodal: queens typically stayed in the nest for either less than 3 min (mean = 52.6 s), between 3 min and 4 h (mean = 27.0 min), or over 4 h (mean = 13.2 h) (Figure A3).

Nest stage significantly predicted foraging activity. Queens in the early foundress stage (i.e., when larvae were present in the nest) took more frequent and shorter foraging trips than they did in the late foundress stage (i.e., around the time adult workers emerged in the nest) (foraging duration LMM *p* = 0.0003; foraging frequency LMM *p* = 0.004). All queens who produced workers (*n* = 7) ceased foraging within approximately 1 week after workers began foraging from the nests (Table A2; Figure 4).

The RFID system was over 95% accurate, with an error rate comparable to in‐person observations. During 66.35 h of simultaneous in‐person and RFID observations, we recorded 152 and 154 instances of a queen entering or leaving a nest via in‐person observations and RFID system recordings, respectively. The RFID system mislabeled or missed 4.6% of observations recorded by in‐person observers (*n* = 5 missed observations; *n* = 2 observations for which the RFID system could not identify the direction of movement). In‐person observers missed 4.5% of observations recorded by the RFID system (*n* = 7).

## 4 Discussion

Natural history information on wild, free‐foraging bumble bee queens is sparse, in part because incipient nests are difficult to locate. We used trap‐nesting and RFID tracking methods to study foundress queens, with the goal of gathering natural history information on the foraging behavior and nest success of wild, foundress queens while developing methods to more intensively study this life stage. We observed frequent, short foraging trips and frequent nest failure among foundress queens in our study. Queens also made fewer, longer trips in the late foundress stage relative to the early stage and ceased foraging entirely within 1 week after workers began foraging from the nest.

Fewer than half of the queens in our study produced adult offspring, and fewer than one quarter of the queens who laid eggs in nest boxes produced gynes at the end of the season, partially because so many queens absconded from nest boxes. This is consistent with the reproductive success rates of trap‐nested colonies in New Zealand (16%; Barron et al. 2000), but lower than those observed in Canada (57%; Richards 1978). We are unable to determine why so many queens absconded in our study, but we suggest that mortality due to the inherent risks of foraging, such as predation, parasitism, or severe weather, may have prevented queens from returning to the nest (Cameron and Sadd 2020; Benoit and Kalisz 2020). It is also possible that queens who absconded did so out of choice and initiated new nests elsewhere, though we think this unlikely because this seems inefficient and would give queens less time for their new colony to grow and reproduce, making them less likely to succeed (Sarro et al. 2021; Malfi et al. 2019; Williams et al. 2009). Ultimately, this failure rate points to the vulnerability of the foundress life stage in bumble bees.

Foundress queens in our study foraged for a shorter duration of time and took a smaller number of trips per day than those reported for wild *B. polaris* queens in the Canadian subarctic (Richards 1978 [30 min, 20 bouts/day]), one of the only studies to quantify wild queen foraging in the past. Queens in our study also foraged for a shorter duration of time than workers of different species, but took a similar or slightly greater number of trips per day than workers (Westphal et al. 2006 [66–82 min, *B. terrestris* ]; Evans et al. 2017 [48 min, 12 bouts/day, *B. terrestris* ]; Hemberger and Gratton 2018 [~30–40 min, *B. impatiens* ]; Minahan and Brunet 2018 [58 min, 6 bouts/day, *B. impatiens* ]; Baur et al. 2019 [32–41 min, *B. huntii* ]). These observed differences may be explained in part by differences in landscape, species, or caste. For example, species differences and life‐history patterns may influence foraging behavior. Subarctic bumble bee queens develop their ovaries and rear their first clutch of offspring faster than those in temperate areas (Vogt et al. 1994), and this accelerated life pace may help explain the observed disparities in foraging activity between *B. polaris* queens and the queens in our study. Due to low sample sizes, we were unable to compare foraging activity among species in our study, though we might expect differences among species, in part due to differences in body size. Larger bee species have been observed to forage farther than smaller species (Greenleaf et al. 2007) and larger bumble bee workers are more efficient foragers and collect more nectar and pollen per trip than smaller workers (Spaethe and Weidenmüller 2002; Goulson et al. 2002); yet there is mixed evidence as to whether these trends extend to other foraging activity patterns such as foraging duration (Spaethe and Weidenmüller 2002; Goulson et al. 2002). The resource environment can also influence foraging patterns. Our study was conducted in a floral‐rich, montane ecosystem, whereas many studies cited above were conducted in more punctuated agricultural or urban environments. The high floral density in our study could have resulted in relatively shorter foraging trips (Hemberger and Gratton 2018).

Independent of species or life history, caste and life stage may further influence foraging activity. As the only adult caretaker in the nest, the demands on a queen's time likely differ from those of workers. For example, previous laboratory studies (albeit on other species) have shown that individual larvae are fed approximately once per hour (Gustilo et al. 2023; Costa et al. 2021), and insufficient feeding or temperature regulation can result in smaller, slower growing larvae or even larval death (Pereboom et al. 2003; Heinrich 2004). Nests with no adult individuals present in them may also be more susceptible to usurpation, predation, and invasion (Korb and Heinze 2008). Thus, short foraging bouts may enable queens to feed and incubate brood more often and leave the nest unattended for shorter periods of time, whereas workers may be able to leave the nest for longer without the same risks. This is also consistent with our observation that queens foraged for shorter periods of time in the early foundress stage, when queens were independently caring for immature larvae, relative to the late foundress stage, around the time that adult workers emerged.

Queens who successfully produced adult workers ceased foraging within days of workers beginning to forage from the nest. When queens stopped foraging, they did so abruptly, with no apparent change in queen foraging behavior in the days or hours leading up to this cessation. This finding is consistent with laboratory work showing that foundress queens are highly sensitive to the emergence of the first workers in the nest and readily reduce parental care behaviors following worker emergence (Shpigler et al. 2013; Woodard et al. 2013; Gustilo et al. 2023). This is hypothesized to allow queens to readjust their time and energy balance to focus on reproduction soon after the first workers appear in the nest (Sarro et al. 2021). In laboratory‐reared, nest‐founding *B. impatiens*, queen food collection behavior appears to be a binary task that queens either do or do not perform, with the number of workers in the nest mediating this transition (Gustilo et al. 2023). The abrupt transition observed in our study is consistent with this binary pattern, in that queens in our study either foraged frequently or not at all. Foraging is an inherently risky behavior because it involves leaving the protection of the nest, and ceasing foraging as soon as workers are capable of carrying out this task may minimize mortality risk for the queen.

We observed additional, curious phenomena in our study that shed light on the biology of wild bumble bee queens. First, we observed queens remaining outside the nest overnight, though bumble bees have poor night vision and do not fly well in the dark (Chittka et al. 1999). It is possible these queens were foraging late in the day and were unable to make it back to their nests before dark, or sudden, inclement weather halted their flight for a number of hours. Whether there may have been alternative reasons for queens to stay out of the nest overnight is yet unknown. Additionally, we observed a conspecific nest invasion, for which we are aware of at least two possible motivations. First, the invading queen may have been attempting to lay eggs in the nest. Conspecific nest usurpation has been documented in bumble bee queens (Richards 1978) and workers (Zanette et al. 2014), and could explain our observations. Alternatively, because the invading queen entered the nest so frequently for only minutes at a time, we suggest the purpose of these invasions may have been to take nectar or pollen from the nest, instead of or in addition to foraging on flowers. Honeypots in the nest provide concentrated food resources and would enable the invading queen to substantially reduce energy expenditure in foraging. Early season nest invasion is rarely documented in wild bumble bees because it is difficult to observe early spring nests (but see Milliron and Oliver 1966; Sakagami and Nishijima 1973; Richards, 1978). Trap nesting provides a valuable opportunity to uncover new information about wild bumble bees during this elusive life stage.

It must be cautioned that our study observed a small number of queens in human‐made nest boxes. Although wild queens chose these boxes as suitable nest sites, they differ from natural nests. It is interesting to note that five queens that initially selected nest boxes absconded prior to laying eggs; this may indicate that the queens found the nest boxes initially attractive but ultimately not suitable as nesting sites. The RFID tags weighed more than a typical worker's pollen load (Naumchik and Youngsteadt 2023), and it is possible they could have interfered with flight or energy balance in our study, especially in small queens. Additionally, our RFID methods did not distinguish between pollen and nectar collecting trips, and this would be an interesting avenue for future expansion. Our RFID records were, however, highly accurate when compared to a human observer and were successful in capturing phenomena that a human observer would likely miss, such as bees remaining outside the nest overnight and multiple conspecific queens in a single nest. These observations underline the power of this trap nesting and RFID‐tracking method as a methodology to monitor foraging in early season nests.

Though our methodology required considerable effort, it ultimately provided valuable and novel insights into bumble bee biology. Despite the significant economic and ecological importance of bumble bees, large portions of their life cycle, including the early nesting stage, remain obscure (USFWS 2021). We show that novel insights into the behavior of nest founding queens can be gained through our methodology and present this trap‐nesting and subsequent RFID tracking method as a promising, albeit resource‐intensive, path forward for studying this evasive, incipient life stage in bumble bees.

## Author Contributions

**Erica Sarro Gustilo:** conceptualization (lead), data curation (lead), formal analysis (lead), funding acquisition (equal), investigation (lead), methodology (lead), project administration (equal), visualization (lead), writing – original draft (lead), writing – review and editing (equal). **William H. Grover:** conceptualization (supporting), funding acquisition (equal), methodology (supporting), resources (lead), software (lead), writing – review and editing (supporting). **S. Hollis Woodard:** conceptualization (equal), funding acquisition (equal), investigation (supporting), methodology (supporting), project administration (equal), resources (equal), writing – original draft (supporting), writing – review and editing (equal).

## Conflicts of Interest

The authors declare no conflicts of interest.

## Data Availability

Raw data and all code associated with filtering and analyses are available on Dryad (https://doi.org/10.5061/dryad.70rxwdc70). Custom printed circuit board designs, Arduino code, and other resources associated with the custom RFID tracking system are available on GitHub (https://github.com/groverlab/RFIDbee).

## Appendix A

**TABLE A1:** Sample sizes for nestbox characteristics.

Characteristics	Alt. 1	Alt. 2	Alt. 3	Alt. 4	Alt. 5	Alt. 6
Landscape type	Aspen forest (36)	Conifer forest (7)	Willow (23)	Open meadow (13)	Cabin edge (18)	Mixed (3)
Box location	< 2 m from edge of Landscape Type (53)	> 2 m from edge of Landscape Type (47)				
Box height	Ground (74)	Tree (26)				
Mouse nest Materials	Present (19)	Absent (81)				
Clay ball	Present (50)	Absent (50)				
RFID box	Attached (21)	Absent (79)				
Plastic sheeting	Attached (14)	Absent (86)				

*Note:* Numbers in parentheses represent sample sizes for each characteristic. “Alt.” columns refer to the alternative states for each characteristic. Rows refer to individual characteristics and are independent from one another. All rows sum to 100 because there were 100 boxes total. To the greatest degree possible, factors were stratified across boxes to avoid multicollinearity (not displayed here; details for each individual box can be found in the published raw data).

**TABLE A2:** Summary of relevant dates and information for all observed queens.

Year	Species and Bee ID	Box Characteristics	Date queen first observed in nest	Stage of offspring on day of tagging	Queen RFID‐tagged; dates of recording	Date workers first observed at nest^a^	Last day queen was recorded leaving nest	Date of last queen sighting before death/absconding, if applicable	Stage of offspring on the day of death/absconding	Did the nest produce gynes?
**2021**	***B. appositus***	**Aspen forest edge; ground; mouse nest material; clay ball**	**June 9**	**Mature larvae**	**June 16–July 26**	**June 28** ^a^	**July 4** ^b^	—	—	**No**
**2021**	***B. centralis***	**Willow edge; ground; clay ball; RFID box attached**	**June 13**	**Young larvae**	**June 14–July 26**	**June 28** ^a^	**July 4**	—	—	**No**
**2021**	***B. rufocinctus*** (1)	**Aspen forest; tree; clay ball**	**June 13**	**Young larvae**	**June 16–July 26**	**July 5** ^a^	**July 3**	—	—	**Yes**
**2022**	***B. rufocinctus*** (2)	**Aspen forest; tree**	**June 22**	**Young larvae**	**June 28–July 30**	**July 22**	**July 28**	—	—	**No**
**2022**	***B. rufocinctus*** (3)	**Conifer forest edge; ground; mouse nest material**	**June 27**	**Mature larvae**	**July 1–July 11**	**July 5**	**July 8**	—	—	**Yes**
**2022**	***B. fervidus*** (1)	**Aspen forest; ground; clay ball**	**June 29**	**7 adult workers + larvae and pupae**	**July 5–July 11**	**Prior to tagging**	**July 9**	—	—	**Yes**
**2022**	***B. fervidus*** (2)	**Conifer forest edge; ground; mouse nest material**	**May 31**	**Unobserved**	**June 7–June 29**	—	**June 25**	**June 25 (absconded)**	**Young larvae**	—
**2022**	***B. fervidus*** (3)	**Cabin edge; ground**	**June 27**	**Mature larvae**	**July 1–July 17**	**July 15**	**July 15**	**July 15** **(absconded; subject to invading queen)**	**Five adult workers + pupae**	—
**2022**	***B. flavifrons***	**Willow edge; ground; clay ball**	**June 13**	**Eggs**	**June 16–July 3**	—	**June 30**	**June 30** **(absconded)**	**Pupae + eggs**	—
2021	*B. rufocinctus*	Aspen forest; ground	June 9	Young larvae	—	—	—	June 13 (died in nest)	Young larvae	—
2021	Unknown	Aspen forest; ground; mouse nest material	June 9	—	—	—	—	June 16 (absconded)	Young larvae	—
2022	*B. appositus*	Aspen forest edge; ground; clay ball	June 29	Young larvae	July 5–July 11	—	—	July 6 (died in nest; subject to invading ant colony)	Young larvae	—
2022	*B. appositus*	Willow stand; ground; mouse nest material; clay ball	July 15	Young larvae	July 16–July 21	—	—	July 17 (absconded)	Young larvae	—
2022	*B. bifarius*	Aspen forest; tree; mouse nest material	June 12	—	June 19–June 23	—	—	June 20 (absconded)	None	—
2022	Unknown	Aspen forest; tree; clay ball	June 5	—	—	—	—	< June 8 (absconded)	None	—
2022	Unknown	Aspen forest; tree; clay ball	June 9	—	—	—	—	< June 13 (absconded)	None	—
2022	Unknown	Mixed forest; tree	June 22	—	—	—	—	< June 28 (absconded)	None	—
2022	Unknown	Aspen forest; tree; clay ball	July 17	—	—	—	—	< July 17 (absconded)	None	—

*Note:* Bolded rows indicate queens for which we successfully tracked foraging behavior. We numerically labeled RFID‐tracked individuals of the same species to enable identification of their corresponding foraging data in previous figures.

^a^ Monitoring for workers in 2021 was not standardized. These dates may be overestimated by up to approximately 7 days.

^b^ This *B. appositus* queen stopped foraging with any regularity after July 4 but was recorded leaving the nest for 288 min on July 13 and 2.6 min on July 18.
